# Duration taken for the anal sphincter pressures to stabilize prior to anorectal manometry

**DOI:** 10.1186/s13104-018-3451-1

**Published:** 2018-06-05

**Authors:** Dakshitha Praneeth Wickramasinghe, Umesh Jayarajah, Dharmabandhu Nandadeva Samarasekera

**Affiliations:** Department of Surgery, Faculty of Medicine, University of Colombo, P.O. Box 271, Kynsey Road, Colombo 8, Sri Lanka

**Keywords:** Anal sphincter pressure, High resolution manometry, Lead-in time, Stabilization time

## Abstract

**Objectives:**

Anorectal manometry (ARM) is an integral part of evaluating the anal sphincter function. The current recommendation of waiting for 5 min (lead-in-time) prior to beginning the recording has no evidence. A prolonged procedure may reduce patient compliance.

**Results:**

We analyzed data from 100 consecutive patients who underwent 3-dimensional ARM at a single center. Their pressure studies were analyzed in consecutive 10-s segments, beginning from the time of insertion of the probe into the anal canal. We defined stabilization of the pressure as the absence of a pressure difference among two consecutive 10-s segments. The study population had 31 males. Their mean age was 33.0 years (SD-14.4). The mean time for the pressure to stabilize was 84.2 s (SD-29.5), range 17.2–203.7 s, 95th percentile 136.2 s. Eleven and one participant(s) took longer than 120 and 150 s for the pressure to stabilize, respectively. There was no correlation of sex (Mann–Whitney U test, p = 0.89) and the time to pressure stabilization. Age and the time to stabilize (Spearman rho − 0.246, p = 0.017) showed a weak negative correlation. A lead-in-time of 5 min, as recommended by present guidelines may be unnecessary. Waiting for 150 s/2½ min may be sufficient and will minimize the procedure duration.

## Introduction

Anorectal manometry (ARM) is one of the commonest methods used to assess the anal sphincter function. The procedure involves insertion of a catheter into the anorectum and connecting it to a pressure recorder to measure the intraluminal pressure. Even though complex procedures and maneuvers had been attempted several decades previously [1], anorectal manometry was first used in patient assessment in the 1980s [2–4]. The initial devices had an intraluminal balloon [5] and afterwards, water perfused [6] and solid state [7] manometers were developed. The initial devices required either pull-through manoeuvres or rotation to assess the entire anal canal because they only had a few transducers. Therefore, they could not acquire the pressures of the entire anal canal simultaneously. The advancement of electronics resulted in the miniaturization of sensors, which allowed more sensors to be placed on the probes. This enabled a much higher number of pressure points to be recorded, resulting in the development of high-resolution anorectal manometry (HRARM) in 2007 [8] and three dimensional (3D) anorectal manometry (3DARM) in 2010 [9]. The latter provides sufficient radial pressure resolution that allows simultaneous circumferential pressure assessment of the high-pressure zone of the anal sphincters. This pressure resolution also makes pull through maneuvers unnecessary, thereby minimizing motion artefacts and other confounders.

ARM provides information about the resting pressure (RP), squeeze pressure (SP) and length of the anal canal (anal high pressure zone length—HPZL) by direct measurement. A balloon attached to the tip of the catheter allows additional measurements such as rectal sensory thresholds and rectoanal inhibitory reflex to be elicited.

Several laboratory manuals and guidelines recommend waiting for 5 min after inserting the probe before taking any pressure measurements [10–12]. One justification for this is the presence of ultra-slow wave activity [12, 13], which might interfere with the interpretation of the resting pressure [10]. However, there is no scientific basis for the duration of the rest period. A prolonged procedure causes discomfort and reduces the patient compliance. Patient compliance is essential for certain maneuvers performed in ARM. The objective of this study was to identify the time taken for the anal sphincter pressures to stabilize following insertion of the pressure transducer.

## Main text

Consecutive patients who underwent 3DARM for a multitude of complaints were included in the study. They were all investigated and treated at the University Surgical Unit of the Faculty of Medicine, University of Colombo, Colombo, Sri Lanka.

Basic demographic details were also recorded at the time of assessment. Patients were assessed without using any bowel preparation but were requested to evacuate the bowel prior to the test.

3D ARM was performed with the patient in the left lateral position. We used the ManoScan AR system by Given Imaging (Yoqneam, Israel). The manometry probe is 10 cm in length and 10.75 mm in diameter. The probe is attached to the amplifier and recording system and the pressure plots are displayed in the proprietary software (Manoview AR, Given Imaging, Yoqneam, Israel). The software linearly interpolate the spaces between the sensors to form a continuous grid.

The probe was inserted into the anal canal after lubricating and positioned to place the high pressure zone (HPZ) in the middle of the pressure sensitive part and the orientation marker at 6 o’ clock. HPZ is defined as the length of the anal canal with a resting pressures at least 30% higher than rectal pressure [14]. The probe was maintained in this position for 5 min and the pressures were recorded continuously. Afterwards, RP (one measurement lasting 20 s) and SP (three attempts for a duration of 20 s each) were assessed. The rectoanal inhibitory reflex (RAIR) and rectal sensation were evaluated if necessary. Atmospheric pressure was the reference point for all values.

The pressure recording was analyzed in consecutive 10-s segments from the moment the probe was entered into the anal canal, up to 5 min. Then, each 30-s segment was compared with the previous 30-s segment visually to identify any differences in pressure. Stabilization was defined as the lack of a difference in pressure values between two consecutive 10- and 30-s segments. If the pressures changed significantly after stabilization, the next/last stabilization was considered for the analysis.

The data were recorded and analyzed using SPSS version 20 (IBM Corp. Released 2011. IBM SPSS Statistics for Windows, Version 20.0. Armonk, NY: IBM Corp.). All continuous data are described with the mean and standard deviation. Mann–Whitney test was used to compare values between the sexes. Correlations were identified using Pearson correlation coefficient (Pearson ρ). The statistical significance was set at p < 0.05.

Data from 100 consecutive patients, including 31 males were included in the analysis. The mean age of the sample was 33.0 (SD-14.4) years. The majority (n = 41) underwent testing as a part of their evaluation of recurrent fistula in ano. The remaining were being investigated for anal incontinence (n = 38) or anal sphincter injuries (n = 21). The latter group included obstetric, impalement and war injuries.

**Fig. 1:**
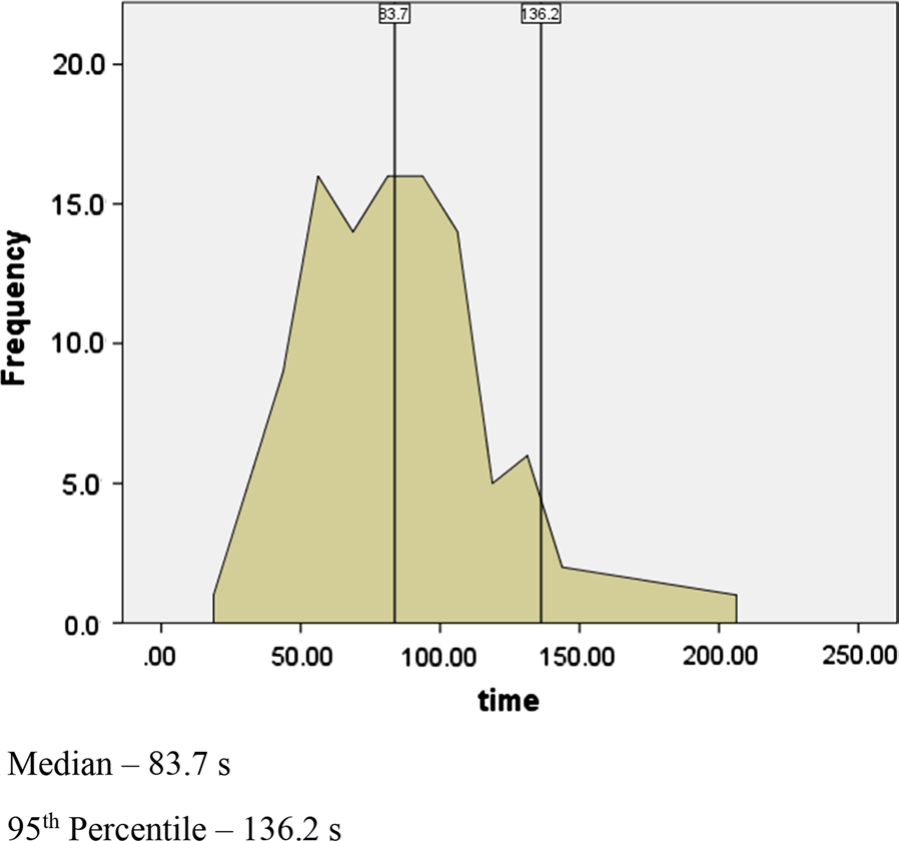
Distribution of the time taken for stabilization

The median time to stabilize was 83.6 (SD-29.5) seconds. The values ranged from 17.2 to 203.7 s, with the 95th percentile being 136.2 s (Table 1 and Fig. 1).

**Table 1:** Time taken for the basal pressure to stabilize

Median	83.6000
Minimum	17.20
Maximum	203.70
Percentiles	
25	61.6250
50	83.6000
75	102.6250

Only 11 participants (11%) took longer than 120 s and 1 participant (1%) longer than 150 s for the pressure to stabilize.

The time to stabilize did not differ with sex (Mann–Whitney U test, p = 0.89). There was a weak negative correlation between age and time to stabilize (Spearman rho − 0.246, p = 0.017). However, upon subgroup analysis, this correlation was only seen among female patients (Spearman rho − 0.322, p = 0.008). There was no correlation between the age and time taken to relax among males (Spearman rho − 0.056, p = 0.779).

In some patients, anorectal manometry can cause pain and discomfort [15]. Evidence from patients who underwent colonoscopy indicates that patients who perceive less pain or discomfort had a higher rate of returning for a repeat assessment [16], suggesting better compliance. Similarly, reducing patient discomfort in ARM will improve patients returning for repeat assessments and their compliance during testing. This is essential in patients with sphincter injuries and sphincter repairs, who require repeated ARM assessments.

Our findings indicate that the traditional 5-min waiting time may be unnecessary. Ninety-nine percent of our patients had their anal canal resting pressure stabilize in under 150 s, which is half the recommended time. A standard ARM assessment of resting and squeeze pressure measurement can often be completed in several minutes. Therefore, if the initial resting time is reduced, the whole procedure can be completed within the present recommendation of resting time of 5 min. Since there were no significant associations between sex or the age, and the time taken for the pressure to stabilize, the waiting time can be recommended for all adult patients, irrespective of their age or sex.

## Limitations

The main limitation of this study is that our sample only contained Asians. However, previous work of the authors indicate that the manometry profile of Sri Lankans are similar to Caucasians [17]. Therefore, the recommendation could be extended to Caucasians. Furthermore, a cross-over design where patients underwent both the “classical” 5-min lead-in and the shorter lead-in may have further strengthened the findings. This study is a preliminary observation study and a larger, randomized study would ensue.

## Abbreviations

3DARM: three dimensional (3D) anorectal manometry
ARM: anorectal manometry
HPZ: high pressure zone
HRARM: high-resolution anorectal manometry
RAIR: rectoanal inhibitory reflex
RP: resting pressure
SP: squeeze pressure

## References

[1] Duthie HL, Watts JM (1965). Contribution of the external anal sphincter to the pressure zone in the anal canal. Gut.

[2] Sakaniwa M (1989). Computerized analysis of anorectal manometry. Prog Pediatr Surg.

[3] Hancke E (1988). Anorectal manometry with the microtransducer. Chirurg.

[4] Vela AR, Rosenberg AJ (1982). Anorectal manometry: a new simplified technique. Am J Gastroenterol.

[5] Schuster MM (1965). Simultaneous manometric recording of internal and external anal sphincteric reflexes. Bull Johns Hopkins Hosp.

[6] Arndorfer RC (1977). Improved infusion system for intraluminal esophageal manometry. Gastroenterology.

[7] Welch RW (1979). Manometry of the normal upper esophageal sphincter and its alterations in laryngectomy. J Clin Invest.

[8] Jones MP, Post J, Crowell MD (2007). High-resolution manometry in the evaluation of anorectal disorders: a simultaneous comparison with water-perfused manometry. Am J Gastroenterol.

[9] Rao SSC (2010). Advances in diagnostic assessment of fecal incontinence and dyssynergic defecation. Clin Gastroenterol Hepatol.

[10] Rao SS (2002). Minimum standards of anorectal manometry. Neurogastroenterol Motil.

[11] Diamant NE (1999). AGA technical review on anorectal testing techniques. Gastroenterology.

[12] Lee TH, Bharucha AE (2016). How to perform and interpret a high-resolution anorectal manometry test. J Neurogastroenterol Motil.

[13] Rao SS (1988). Anorectal contractility under basal conditions and during rectal infusion of saline in ulcerative colitis. Gut.

[14] Lowry AC (2001). Consensus statement of definitions for anorectal physiology and rectal cancer. Colorectal Dis.

[15] Szojda MM (2008). Referral for anorectal function evaluation is indicated in 65% and beneficial in 92% of patients. World J Gastroenterol.

[16] Redelmeier DA, Katz J, Kahneman D (2003). Memories of colonoscopy: a randomized trial. Pain.

[17] Wickramasinghe DP (2015). Three-dimensional anorectal manometry findings in primigravida. Dig Dis Sci.

